# A prognostic 4-gene expression signature for patients with HER2-negative breast cancer receiving taxane and anthracycline-based chemotherapy

**DOI:** 10.18632/oncotarget.21872

**Published:** 2017-10-17

**Authors:** Pu Cheng, Zhen Wang, Guoming Hu, Qi Huang, Mengjiao Han, Jian Huang

**Affiliations:** ^1^ Department of Surgical Oncology, Second Affiliated Hospital and Cancer Institute (Key Laboratory of Cancer Prevention & Intervention, National Ministry of Education, Provincial Key Laboratory of Molecular Biology in Medical Sciences), Zhejiang University School of Medicine, Hangzhou, China; ^2^ Department of General Surgery (Breast and Thyroid Surgery), Shaoxing People’s Hospital, Shaoxing Hospital of Zhejiang University, Zhejiang, China; ^3^ Department of Medical Oncology, Key Laboratory of Biotherapy in Zhejiang, Sir Runrun Shaw hospital, Medical School of Zhejiang University, Hangzhou, China; ^4^ Gastroenterology Institute, Zhejiang University School of Medicine, Hangzhou, China

**Keywords:** breast cancer, signature, prognosis, relapse, chemotherapy

## Abstract

Breast cancer is a heterogeneous group of diseases with diverse clinicopathological and molecular features. At present, chemo-resistance still poses a major obstacle to successful treatment of HER-2 negative breast cancer. Reliable biomarkers are urgently needed to accurately predict the therapeutic sensitivity and prognosis of such patients. In this study, we identified 3145 distant relapse–free survival (DRFS) associated genes in 310 patients with HER-2 negative breast cancer receiving taxane and anthracycline-based chemotherapy in the GSE25055 dataset using univariate survival analysis. Four genes (SRPK1, PCCA, PRLR and FBP1) were further selected by a robust likelihood-based survival model. A risk score model was then constructed with the regression coefficients of the four signature genes. Patients in the training set were successfully divided into high- and low-risk groups with significant differences in DRFS between the two groups. The predictive value was further validated in GSE25065 dataset and similar results were observed. Moreover, the 4-gene signature was proved to have superior prognostic power compared with several clinical signatures such as tumor size, lymph node invasion, TNM stage and PAM50 signature. Our findings indicated that the 4-gene signature was a robust prognostic marker with a good prospect of clinical application for HER-2 negative breast cancer patients receiving taxane-anthracycline combination therapy.

## INTRODUCTION

Breast cancer is one of the most common cancers and the second leading cause of mortality for women worldwide [1]. Almost one of eight to ten women will suffer breast cancer during their lifetime [2]. Incidence rate of breast cancer has been on the increase for several years and the average onset age is dropping, which probably occasioned by the changes of lifestyle, environment and the development of screening methods [3–5]. Recent advances of the chemotherapy, radiotherapy, hormone therapy and immuno-biological therapy have dramatically improved the survival for patients with breast cancer. Nevertheless, great individual differences have been found in the outcomes of breast cancer treatments due to the tumor heterogeneity.

Chemotherapy is the chief means of HER2-negative breast cancer treatment, among which, taxane-anthracycline combination regimens have been advised as standard neoadjuvant and adjuvant strategies [6]. At present, *chemo-resistance* still poses a major obstacle to successful treatment of breast cancer, with a lot of patients being under- or over-treated. Though great efforts have been made on the development of effective prognostic indicators through molecular and cell biological studies, outcomes of patients with breast cancer are still predicted largely on the basis of conventional clinicopathological and molecular prognostic factors [7–9]. However, quite a few patients show distinct responses to chemotherapy even if they have same or similar clinicopathological characteristics. Thus, there is a critical need for innovative biomarkers to accurately predict the therapeutic sensitivity and prognosis of HER-2 negative breast cancer.

In this study, we first performed univariate survival analysis and identified 3145 distant relapse–free survival (DRFS) associated genes in 310 patients with HER-2 negative breast cancer from the GSE25055 dataset. After that, a 4-gene prognostic signature was developed by using robust likelihood-based survival model and unsupervised hierarchical clustering analysis. A risk score model was then built by multivariate survival analysis and the prognostic value was further validated in GSE25065 dataset. Our findings suggested that this 4-gene signature could serve as an effective biomarker to predict the chemosensitivity and prognosis for patients with HER-2 negative breast cancer receiving taxane/anthracycline-based therapy.

## RESULTS

### Identification of differentially expressed genes associated with prognosis in the training dataset

**Figure 1:**
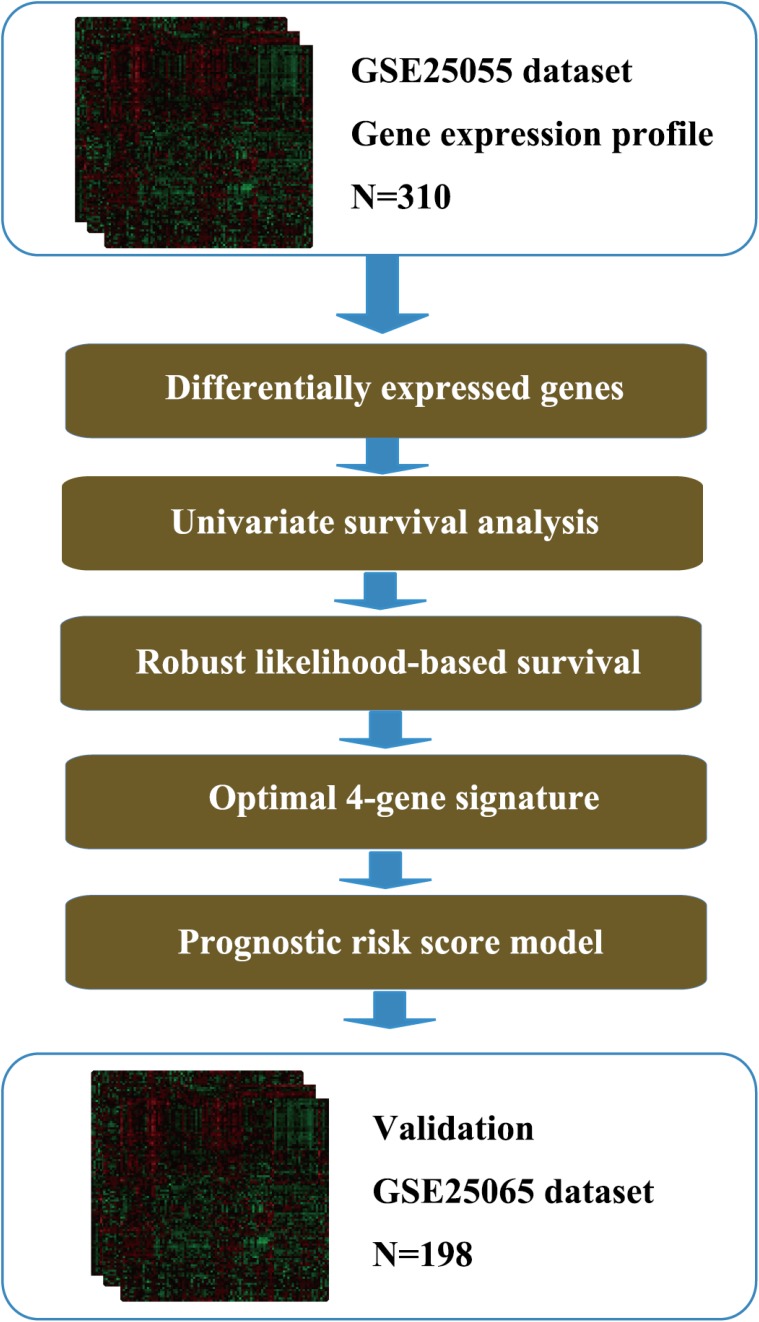
Flow diagram of methods for developing the prognostic 4-gene signature

The overall flow diagram of present study was summarized in Figure 1. The 310 breast cancer samples with expression values of 22283 genes were acquired from the GSE25055 dataset. All the patients were diagnosed HER-2 negative and treated with taxane and anthracycline-based chemotherapy. 13510 differentially expressed probes were selected for further analysis according to the screening criteria described in the Materials and Methods part (Supplementary Table 1). A univariate survival analysis was conducted using Cox proportional hazard regression model based on the expression level of these genes. Finally, 3145 seed genes significantly associated with DRFS (*p* < 0.05) were identified (Supplementary Table 2). The top 20 genes with most remarkable changes are listed in Table 1.

**Table 1:** The top 20 genes with most remarkable changes in training set

Probe ID	Gene symbol	*p* value
211110_s_at	AR	1.39E–08
210476_s_at	PRLR	5.21E–08
200810_s_at	CIRBP	1.24E–07
219648_at	MREG	1.31E–07
202171_at	VEZF1	1.38E–07
205862_at	GREB1	1.54E–07
212811_x_at	SLC1A4	1.87E–07
208935_s_at	LGALS8	2.18E–07
221874_at	KIAA1324	2.36E–07
205428_s_at	CALB2	2.38E–07
203860_at	PCCA	3.16E–07
202200_s_at	SRPK1	4.53E–07
214552_s_at	RABEP1	5.15E–07
205597_at	SLC44A4	5.24E–07
206401_s_at	MAPT	5.92E–07
201951_at	ALCAM	6.67E–07
209696_at	FBP1	7.44E–07
212095_s_at	MTUS1	8.02E–07
218692_at	SYBU	8.11E–07
208682_s_at	MAGED2	9.01E–07

**Figure 2:**
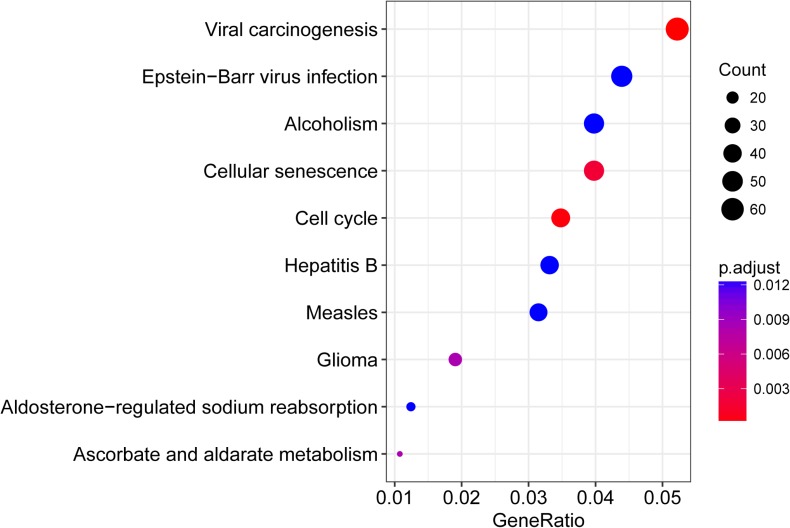
The top 10 enriched pathways for 3145 seed genes significantly associated with DRFS

In order to investigate the main function of the aforesaid seed genes, we performed KEGG pathway enrichment analysis using clusterProfiler. The result showed that these genes were enriched in several key cancer-related signaling pathways such as cell cycle, cellular senescence, pathways in cancer (Supplementary Table 3). The top 10 pathways were shown in Figure 2.

### Development of the 4-gene signature for prognosis prediction in the training dataset

Given the difficulty in using such a large number of genes for clinical diagnosis, we next screened the optimal survival-associated signature genes by a robust likelihood-based survival model. Four genes (SRPK1, PCCA, PRLR and FBP1) were selected as signature genes that can optimally predict the DRFS of patients in the training dataset, as shown in Table 2. KEGG pathway functional annotation was then adopted to explore the function of these four signature genes. As shown in Table 3, these four genes involved in several signaling pathways related to the development and progression of breast cancer.

**Table 2:** Survival-associated gene signature screening using forward selection

Probe ID	Gene symbol	nloglik	AIC
202200_s_at	SRPK1	342.16	686.33^*^
203860_at	PCCA	335.30	674.60^*^
210476_s_at	PRLR	329.94	665.88^*^
209696_at	FBP1	328.87	665.75^*^
212956_at	TBC1D9	328.87	667.73
206401_s_at	MAPT	327.93	667.86
214552_s_at	RABEP1	327.58	669.16
208682_s_at	MAGED2	326.85	669.71
212492_s_at	KDM4B	326.82	671.65
212811_x_at	SLC1A4	323.86	667.71
200670_at	XBP1	323.71	669.42
211110_s_at	AR	322.83	669.65
205597_at	SLC44A4	322.46	670.93
221874_at	KIAA1324	322.38	672.77
219197_s_at	SCUBE2	322.00	673.99
200810_s_at	CIRBP	321.74	675.48
219648_at	MREG	321.03	676.06
205862_at	GREB1	320.37	676.73
202171_at	VEZF1	316.68	671.35

**Table 3:** Results of function annotation analysis for 4 signature genes

KEGG pathway	Gene symbol
Pentose phosphate pathway	FBP1
Fructose and mannose metabolism	FBP1
Glycolysis/Gluconeogenesis	FBP1
Glucagon signaling pathway	FBP1
AMPK signaling pathway	FBP1
Insulin signaling pathway	FBP1
Glyoxylate and dicarboxylate metabolism	PCCA
Propanoate metabolism	PCCA
Valine, leucine and isoleucine degradation	PCCA
Carbon metabolism	PCCA/FBP1
Prolactin signaling pathway	PRLR
Jak-STAT signaling pathway	PRLR
Cytokine-cytokine receptor interaction	PRLR
Neuroactive ligand-recptor interaction	PRLR
PI3K-Akt signaling pathway	PRLR
Herpes simplex infection	SRPK1

**Figure 3:**
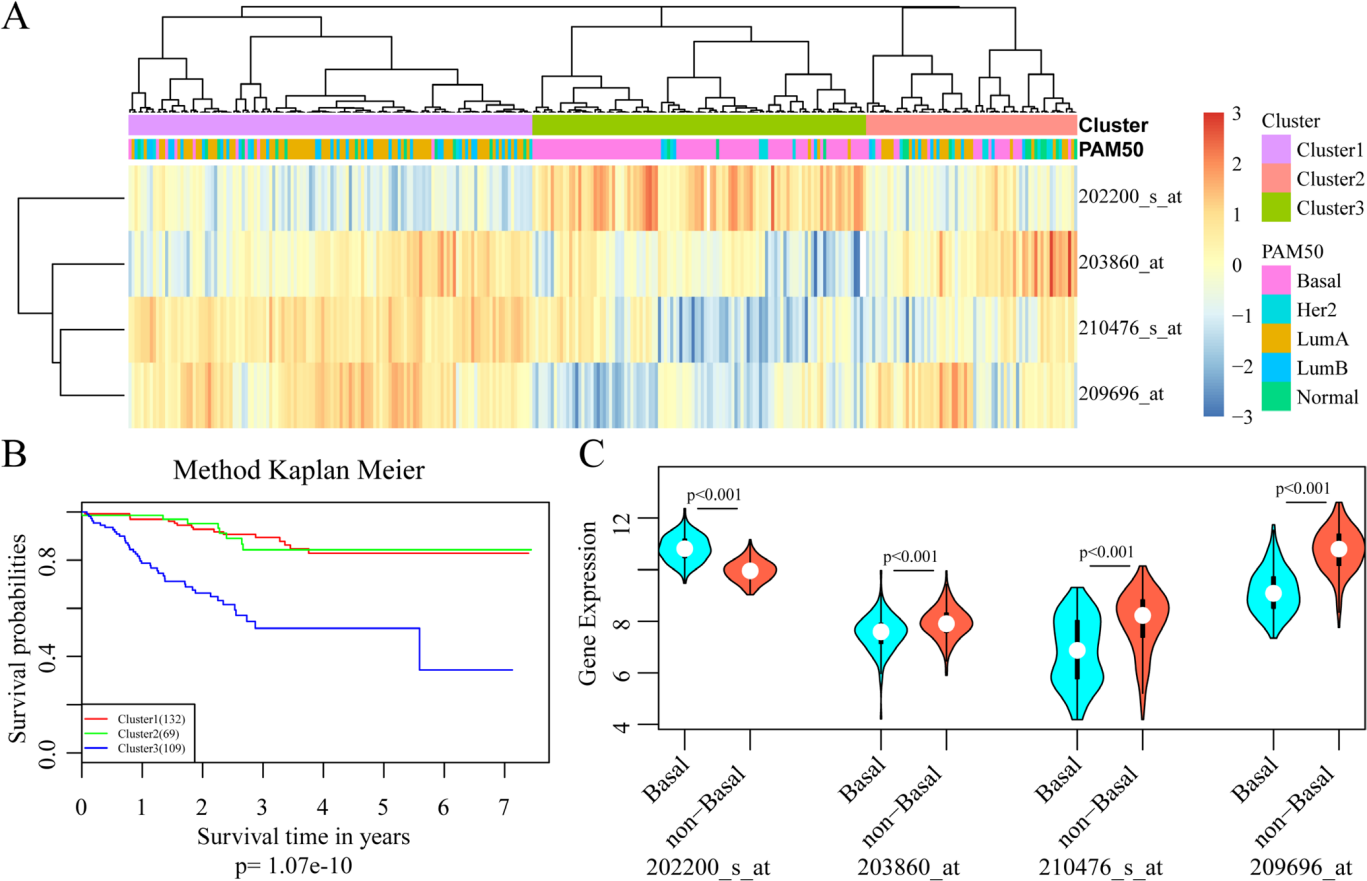
Development of the 4-gene signature for prognosis prediction (**A**) Results of unsupervised hierarchical clustering analysis based on the expression levels of the four signature genes. (**B**) Kaplan–Meier curves for patients in different clusters. (**C**) The mRNA expression of four signature genes in Basal-like and non-basal-like patients.

With the selected gene signature, unsupervised hierarchical clustering analysis was carried out, and the patient population was divided into three sub-classes (Cluster 1, Cluster 2 and Cluster 3), with 132, 69 and 109 samples respectively (Figure 3A). As depicted in Figure 3B, comparing with the other two sub-classes, patients in cluster 3 had much worse outcomes (*p* = 1.07e–10). A closer look at the clinical characteristics revealed that patients in cluster 3 were mostly basal-like subtype (86.2%, 94/109), and the number was only 39.4% in the whole cohort. Thus, basal-like breast cancer was distributed mainly in cluster 3, consisting with the well-known fact that the triple negative patients always had poor outcomes in clinical practice. Moreover, SRPK1 expression was significantly elevated in triple negative samples while PCCA, PRLR and FBP1 expressions were decreased dramatically (Figure 3C).

All these results suggested that this 4-gene signature may have important application in predicting the prognosis for patients with HER-2 negative breast cancer receiving taxane and anthracycline combination regimens.

### Construction and assessment of prognostic risk score model based on 4-gene signature

**Figure 4:**
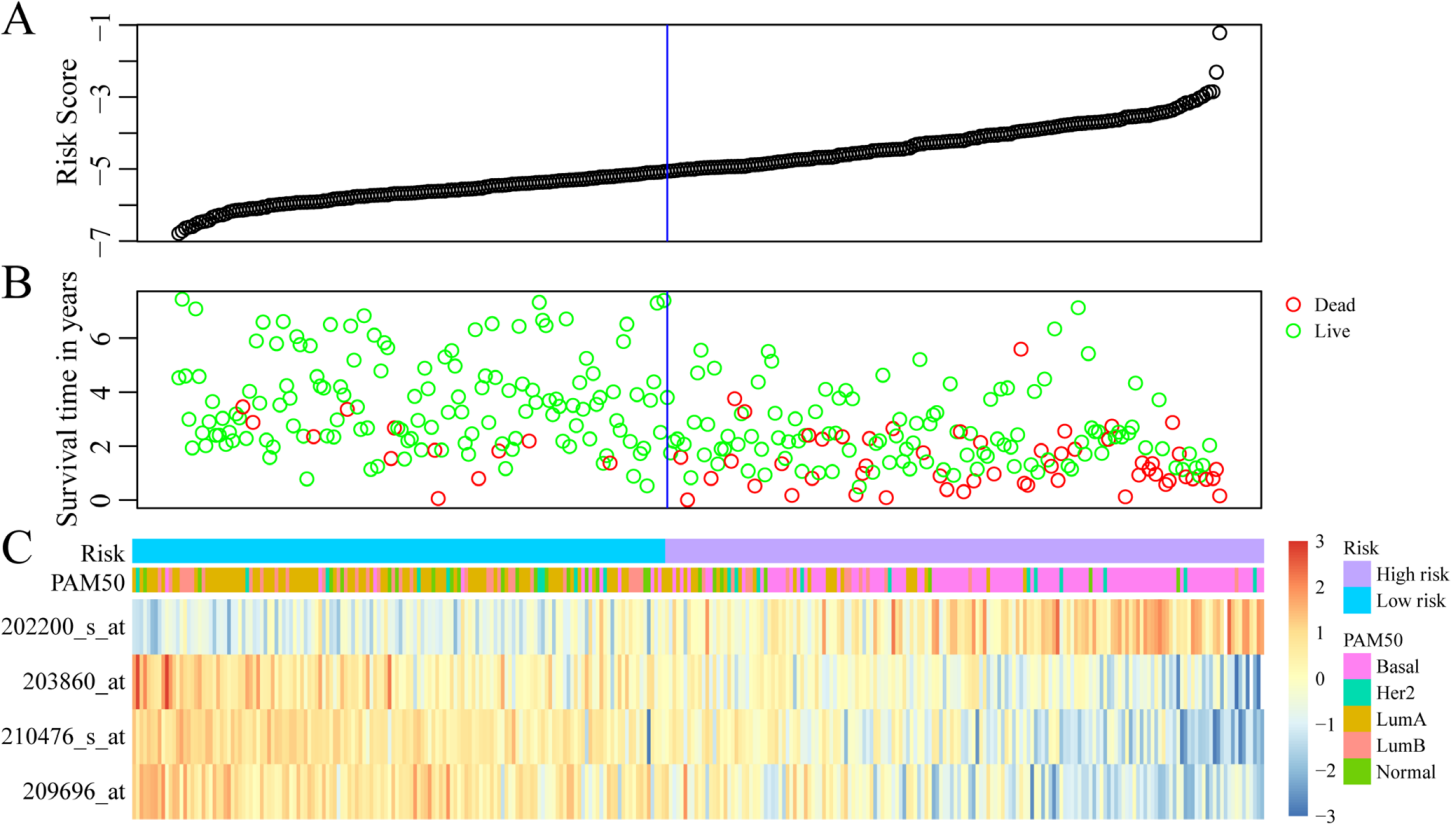
Construction of prognostic risk score model based on 4-gene signature (**A**) The risk score of each sample. (**B**) Different survival status according to the risk score. (**C**) The mRNA expression of four signature genes in each sample with different risk score.

**Figure 5:**
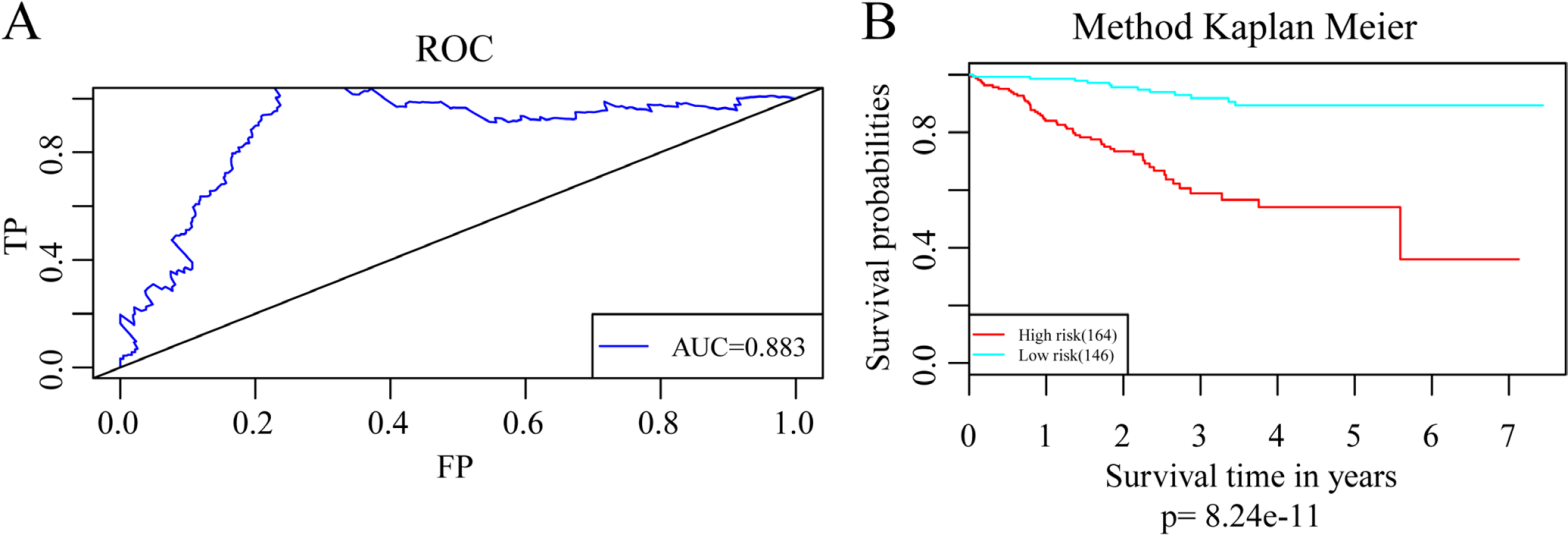
Assessment of the the prediction power of the risk model (**A**) The ROC curve for survival predictions with an AUC of 0.883. (**B**) Kaplan–Meier curves for patients in high and low-risk groups divided with the optimal cut-off score.

The regression coefficients of the four signature genes were generated by multivariate survival analysis. A risk score model was then built as follow: Risk Score = 0.38*exp (SRPK1)-0.56*exp (PCCA)-0.3*exp (PRLR)-0.22*exp (FBP1). With the risk score of each sample, the prognostic differences was evaluated (Figure 4A). As illustrated in Figure 4B, higher risk score indicated greater mortality risk for patient with HER-2 negative breast cancer. We also observed that along with the increase of risk score, the expression level of SRPK1 was up-regulated while the other three were declined (Figure 4C). Receiver operating characteristic (ROC) curve analysis was performed to evaluate the prediction power of the risk model. The area under ROC curve (AUC) was 0.883, indicating good performance of this model for prognosis prediction (Figure 5A). The 310 patients were divided into high and low-risk groups using the optimal cut-off score. As showed in Figure 5B, the DRFS of patients in the high-risk group was significantly shorter than that of the low-risk group (*p* = 8.24e–11).

**Figure 6:**
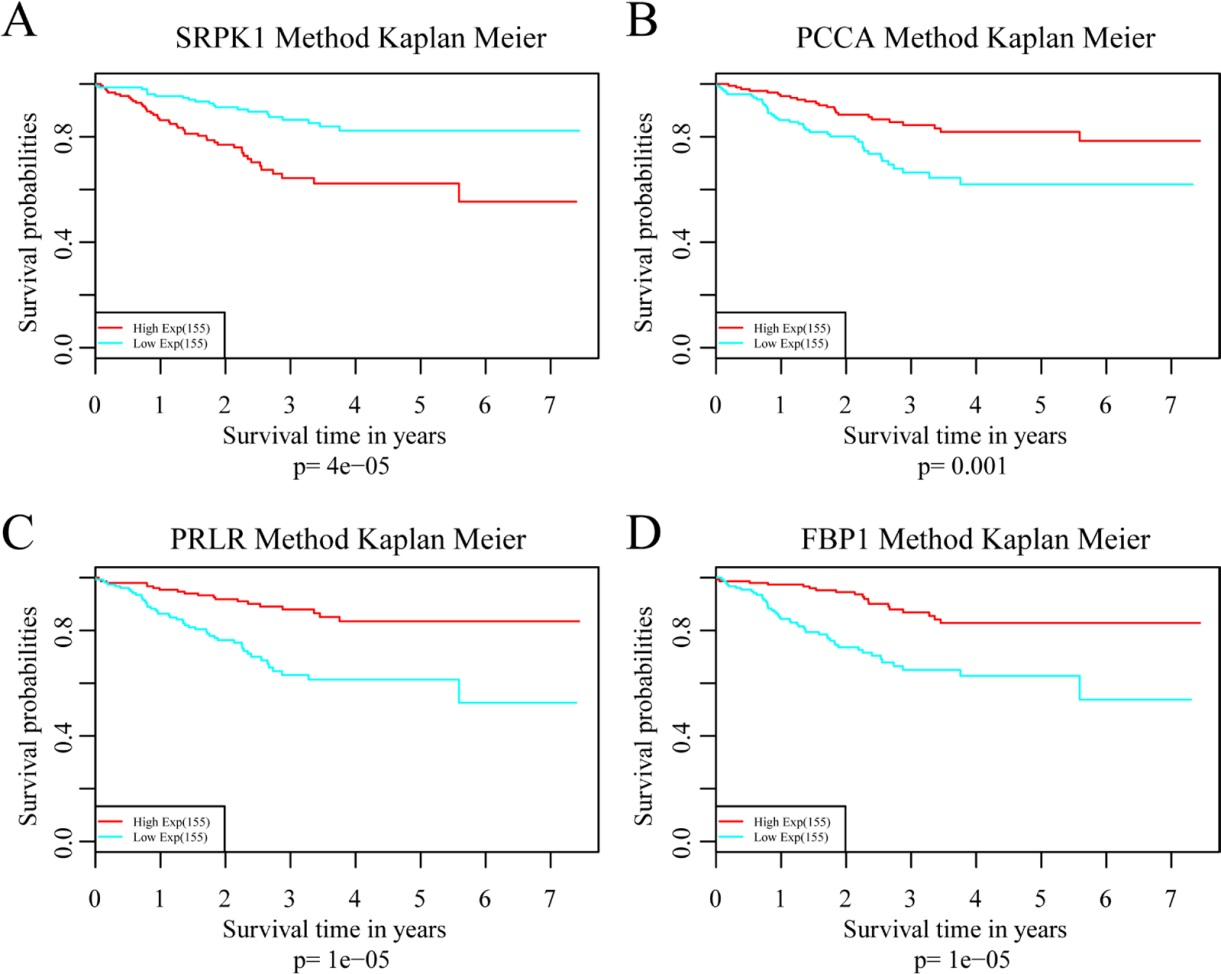
Analysis of the prognostic impact of each single signature gene (**A**) Kaplan–Meier curves for patients in SRPK1 high and low-expression groups. (**B**) Kaplan–Meier curves for patients in PCAA high and low-expression groups. (**C**) Kaplan–Meier curves for patients in PRLR high and low-expression groups. (**D**) Kaplan–Meier curves for patients in FBP1 high and low-expression groups.

**Figure 7:**
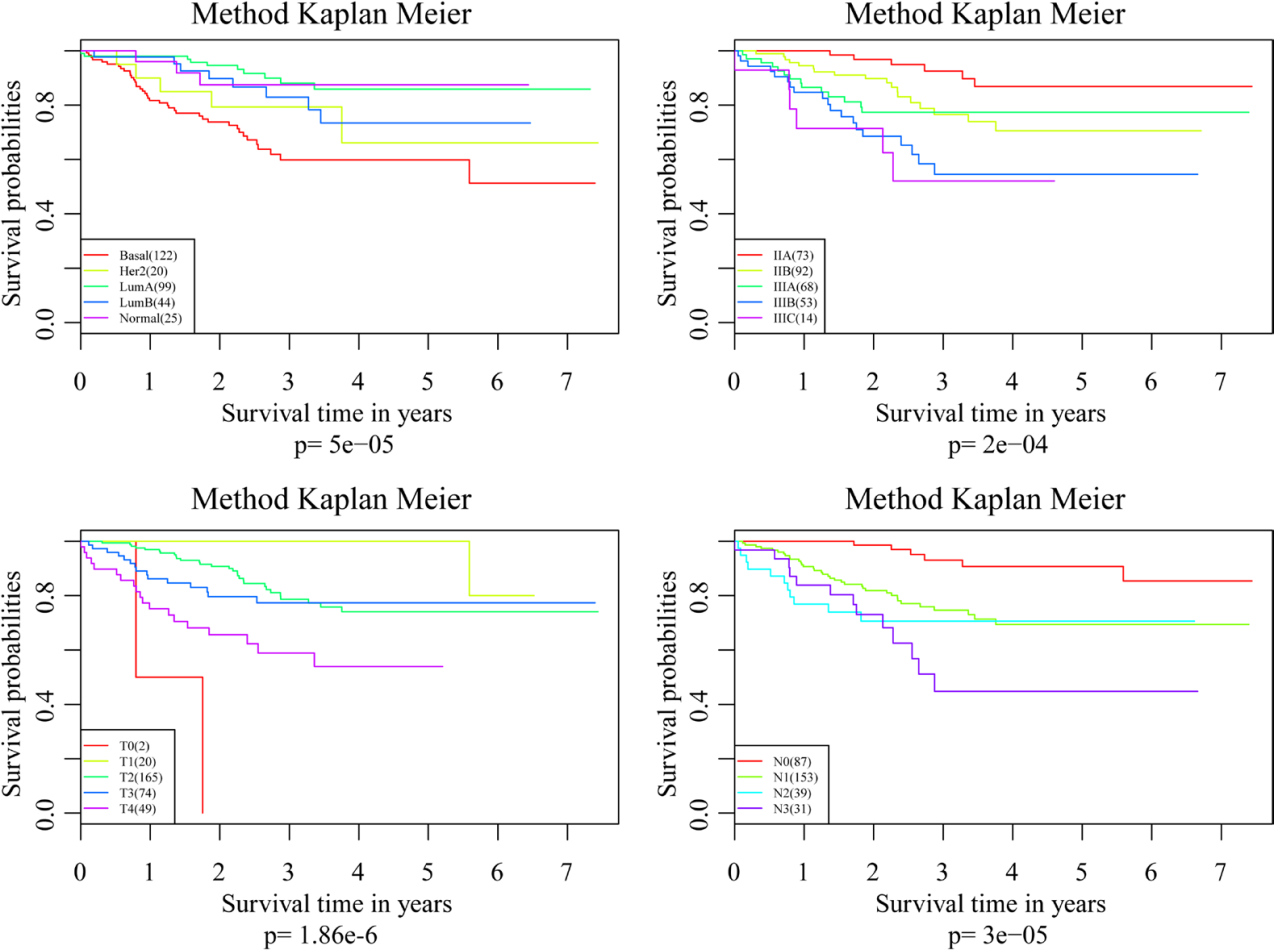
Comparisons of the prediction capacity between 4-gene signature and other clinical classification systems

To investigate the impact on prognosis of each single signature gene, patients of the training dataset were classified into different groups using the median expression level as the threshold. The results of Kaplan-Meier analysis showed that each of the genes had predictive ability on DRFS, especially PRLR and FBP1. However, the prognostic power was found much stronger when these four genes used in combination (Figure 6). We further compared the prediction capacity of this risk score model with other clinical classification systems including tumor size (T), lymph node invasion (N), TNM stage and PAM50 signature. As shown in Figure 7, the 4-gene signature was proved to have the most robust prognostic power among these clinical signatures.

### External validation of 4-gene signature

**Figure 8:**
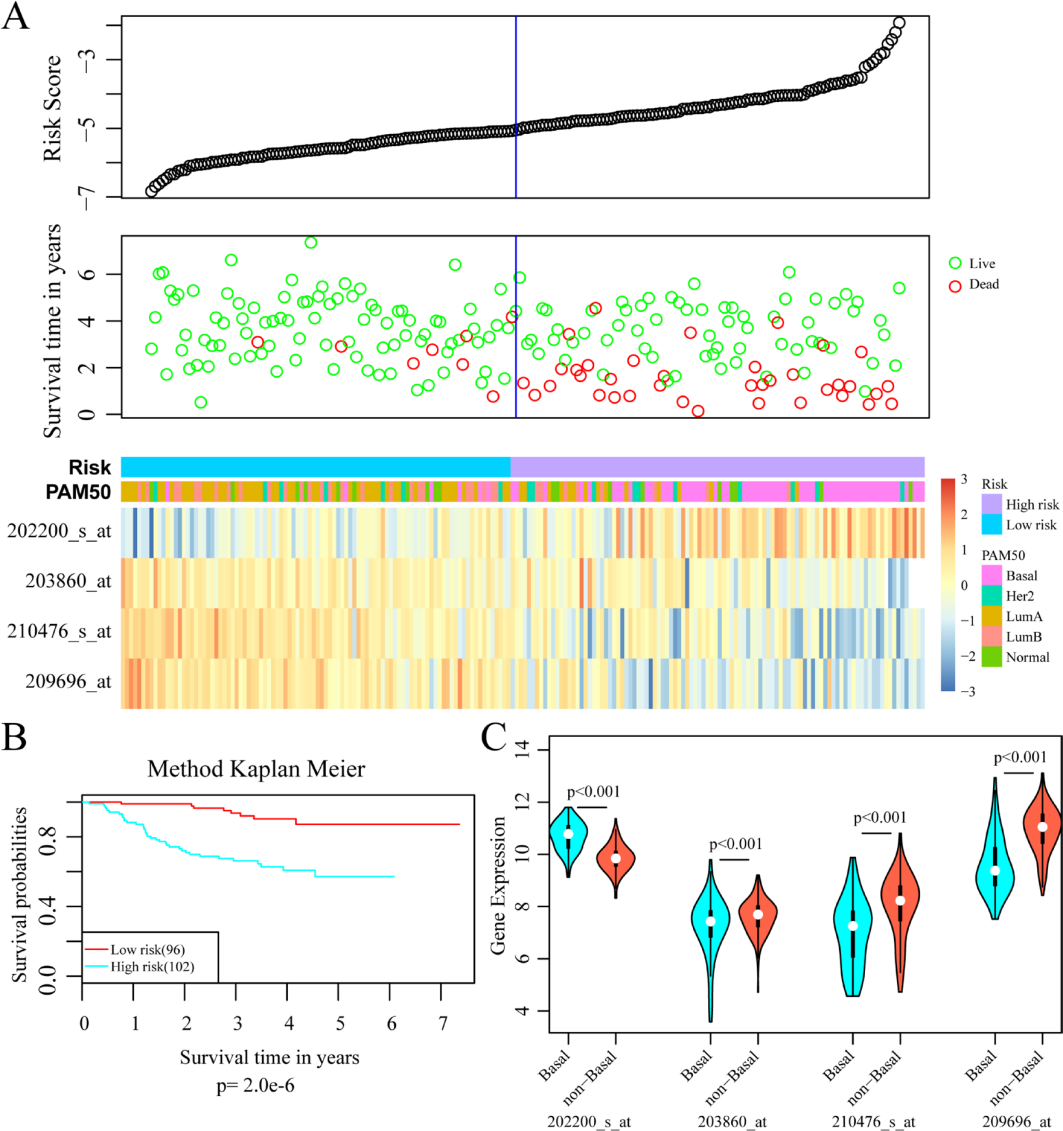
External validation of 4-gene signature (**A**) *Top*: The risk score of each sample in validation set; *Middle*: Corresponding survival status of each sample; *Bottom*: The mRNA expression of four signature genes in each sample with different risk score. (**B**) Kaplan–Meier curves for patients in high and low-risk groups. (**C**) The mRNA expression of four signature genes in Basal-like and non-basal-like patients.

To validate the accuracy and repeatability of the prognostic 4-gene signature, the risk score model was applied to the GSE25065 dataset (*n* = 198). Like that of GSE25055, all the samples of GSE25065 were HER-2 negative following taxane and anthracycline chemotherapy. The prognostic risk score of each patient was then calculated according to the formula. As in the training dataset, higher risk score indicated greater mortality risk for patient with HER-2 negative breast cancer (Figure 8A). Besides, both the expression levels of four signature genes and the proportion of basal-like breast cancer in the GSE25065 dataset were in line with that in the training dataset. The samples were further divided into high-risk group and low-risk group based on the optimal cut-off risk scores. The Kaplan-Meier univariate analysis indicated a statistical significance on DRFS between the two groups (*p* = 2.0e–6, Figure 8B). Furthermore, increased expression of SRPK1 and reduced expressions of the other three genes in basal-like breast cancer were also observed in the validation dataset. Therefore, this 4-gene signature was an effective marker in predicting prognosis for patients with HER-2 negative breast cancer following taxane and anthracycline-based chemotherapy.

## DISCUSSION

Breast cancer is a heterogeneous group of diseases with diverse clinicopathological features and gene dysregulations [10, 11]. Despite the rapid development of therapeutic approaches, there are still many patients suffer from tumor recurrence and metastasis, which is mainly caused by chemo-resistance. Conventional clinicopathological and molecular prognostic factors, such as TNM stage, histological grade, expression of the oestrogen and progesterone receptors, can not effectively estimate the benefits of chemotherapy in HER-2 negative breast cancer. Additionally, tests designed for molecular classification or prognosis without chemotherapy were found lacking of clinical usefulness in the prediction of survival outcomes in chemosensitive patients [12–14]. Therefore, reliable prognostic factors are urgently needed for HER-2 negative breast cancer patients treated with chemotherapy.

Various genetic changes have been found to play important roles in breast cancer initiation and progression [15, 16]. For example, women with pathogenic variants in breast cancer 1 (BRCA1) and BRCA2 were reported to have a cumulative lifetime risk of developing breast cancer between 41% and 90% [17, 18]. Besides, mutations in tumor protein p53 (TP53) [19], phosphatase and tensin homolog (PTEN) [20], serine/threonine kinase 11 (STK11), cadherin 1 (CDH1) [21], partner and localizer of BRCA2 (PALB2) [22] and checkpoint kinase 2 (CHEK2) [23] were also associated with an increased risk of breast cancer. However, there are currently few systemic evaluations on clinical application of these genes as most studies only focused on one or a few genes. In recent decades, high-throughput genomic technologies, such as DNA microarrays and next-generation sequencing, have been widely applied in the studies of cancer heterogeneity. Several risk models have been constructed to predict tumor metastasis, recurrence, treatment response and prognosis by using miRNA [24, 25] and lncRNA expression profiling [26–28].

In the current study, we developed a robust 4-gene signature (SRPK1, PCCA, PRLR and FBP1) to predict DRFS for patients with HER2-negative breast cancer receiving chemotherapy by analyzing the publicly available gene expression profiles from the GEO database.

The GSE25055 dataset was used as training set and total 3145 genes were identified to be significantly associated with DRFS. Results of KEGG analysis revealed that these genes were enriched in several key cancer-related signaling pathways such as cell cycle, cellular senescence, pathways in cancer. We eventually selected four genes (SRPK1, PCCA, PRLR and FBP1) as signature genes for prognosis prediction by using robust likelihood-based survival model. Among which, PCCA was predicted to be associated with energy metabolism. However, there has been no reported study in breast cancer so far. SRPK1, a protein kinase that specifically phosphorylates serine/arginine-rich (SR) splicing factors, has been reported to be involved in a number of biological and pathological processes [29]. Studies have found that SRPK1 expression was up-regulated in breast cancer, which correlated with poor outcome and preferential metastasis to the lungs and brain [30, 31]. Targeted inhibition of SRPK1 may exert some of its antitumor effects in breast cancer through altering the splice pattern and sensitivity to apoptotic signals [32, 33]. PRLR is a type 1 cytokine receptor that has been implicated in the pathology of breast cancer. Emerging evidence suggests that targeting the PRLR signaling pathway may represent a novel antihormonal approach for the treatment of breast cancer [34, 35]. FBP1, the rate-limiting enzyme in gluconeogenesis, is a critical modulator in breast cancer progression by altering glucose metabolism [36, 37]. Recent studies have demonstrated that low or absent expression levels of FBP1 was a critical oncogenic event in epithelial-mesenchymal transition and might be associated with reduced disease-free survival in basal-like breast cancer [38, 39]. Therefore, all the four signature genes may play key roles in the development and progression of breast cancer and are worth further investigation.

The 4-gene signature was first assessed in the training set. Results of ROC analysis showed robust prognostic power with an AUC of 0.883. Patients in the training set were successfully divided into high- and low-risk groups with significant differences in DRFS between the two groups. The predictive value was further validated in another GEO dataset and similar results were observed. Moreover, the 4-gene signature was proved to have superior prognostic power compared with several clinical signatures such as tumor size, lymph node invasion, TNM stage and PAM50 signature. These results indicated that this 4-gene signature was an effective prognostic predictor for patients with HER-2 negative breast cancer following taxane and anthracycline-based chemotherapy.

High-throughput genomic studies have provided new insights into the molecular mechanisms of breast cancer. However, the clinical applicability of this technology was restricted by the cost and information overflow. The 4-gene prognostic signature obtained from our study can overcome this hurdle to some extent. Considering the limited sample size of our study, large-scale cohort studies will be performed in the future to evaluate the prognostic value of this 4-gene signature. In addition, the biological functions of these four signature genes in breast cancer metastasis have not been fully revealed. Thus, further experimental studies should be conducted to uncover the detailed effects of these four genes on the biological behavior and pathogenesis of breast cancer.

In conclusion, our findings demonstrated that the 4-gene signature was a promising prognostic indicator with a good prospect of clinical application for HER-2 negative breast cancer patients receiving taxane-anthracycline combination therapy.

## MATERIALS AND METHODS

### Microarray data acquisition and processing

Two independent breast cancer gene expression profile datasets on the Affymetrix Human Genome U133A platform with corresponding clinical information were downloaded from the publicly available GEO database. All the patients were diagnosed HER-2 negative and treated with taxane and anthracycline-based chemotherapy. The GSE25055 dataset containing 310 samples was used as training set to construct risk model and the GSE25065 dataset containing 198 samples was used as validation set to confirm the prognostic power of the model. The MAS5.0 signal intensity for each probe was log2 transformed and quantile normalized to obtain equal distributions.

### Four-gene signature identification

Differentially expressed probes were preliminary picked out with the criteria as follows: (i) the median expression level of A gene in each sample is 20% higher than that of the whole genome; (ii) the expression level variance of A gene in each sample is 20% higher than that of the whole genome. A univariate Cox proportional hazard regression survival analysis was conducted by using the R package “survival” to obtain the prognosis-related seed genes with *p* < 0.05. KEGG Pathway enrichment analysis was performed to investigate the functions of these seed genes by using the clusterProfiler package in R [40]. A robust likelihood-based survival modeling approach [41–43] was used to select the optimal survival-associated gene signature by using the R package “rbsurv”. The detailed algorithmic procedure is as follows: (i) The samples were randomly split into the training set with N*(1 − p) samples and the validation set with N*p samples (*p* = 1/3). The parameter estimate for each gene was obtained after fitting to the training sample set. With the parameter estimate, log likelihood was computed in both sample sets; (ii) The above procedure was repeated 10 times and the best gene with the largest mean log likelihood was first picked out; (iii) By evaluating every two-gene model, the one with the largest mean log likelihood was selected as the next most appropriate gene. Such forward gene selection process was continued and eventually generated a set of different candidate models; (iv) Akaike information criterion (AIC) statistics was applied for all the candidate models generated in the previous steps. Finally, an optimal model with the smallest AIC was obtained. KEGG pathway functional annotation was further adopted to explore the function of these four signature genes.

### Unsupervised hierarchical clustering and multivariate survival analysis

By unsupervised hierarchical clustering analysis, samples were divided into three sub-classes according to the expression levels of four signature genes [44]. Prognostic differences between these sub-classes were further analyzed with Kaplan-Meier survival analysis [45].

### Prognostic risk score model construction and external data validation

The regression coefficients of the four signature genes were generated by multivariate survival analysis. A prognostic risk score model was constructed to evaluate the effects of this 4-gene signature on prognosis. The risk score of each patient was calculated according to the formula as follow: Risk Score = 0.38*exp (SRPK1)-0.56*exp (PCCA)-0.3*exp (PRLR)-0.22*exp (FBP1). Receiver operating characteristic (ROC) curve analysis was performed to evaluate the prediction power of the risk model by R package “survivalROC” [46] and the area under the curve (AUC) was calculated. The optimal threshold for risk classification based on ROC curve was obtained. The risk score model was applied to the GSE25065 dataset to validate the accuracy and repeatability of the prognostic 4-gene signature.

## SUPPLEMENTARY MATERIALS TABLES








