# Supplementary material for: A prognostic 4-gene expression signature for patients with HER2-negative breast cancer receiving taxane and anthracycline-based chemotherapy
Source: Oncotarget. 2017 Oct 17;8(61):103327–39. doi: 10.18632/oncotarget.21872 (PMC5732731; doi:10.18632/oncotarget.21872)
Supplement: Supplementary file 1 [file oncotarget-08-103327-s001.pdf]

A prognostic 4-gene expression signature for patients with HER2-negative breast cancer receiving taxane and anthracycline-based chemotherapy

SUPPLEMENTARY MATERIALS

Supplementary Table 1: 13510 differentially expressed probes in training set. See Supplementary_Table_1

Supplementary Table 2: 3145 distant relapse-free survival (DRFS) associated genes in 310 patients from the GSE25055 dataset. See Supplementary_Table_2

Supplementary Table 3: Results of KEGG pathway enrichment analysis for 3145 seed genes significantly associated with DRFS. See Supplementary_Table_3
